# Mechanical Reinforcement and Early-Stage Deterioration of Cellulose Nanocrystal-Reinforced Isosorbide-Based Polycarbonate Nanocomposites Under Humid Soil Exposure

**DOI:** 10.3390/polym18212751

**Published:** 2026-11-05

**Authors:** Yuli Nurul Maulida, Ellana Nabilah Nur Averina Ansar, Merreta Noorenza Biutty, PilHo Huh, Seong Il Yoo

**Affiliations:** 1 Department of Polymer Engineering, Pukyong National University, Busan 48547, Republic of Korea; 2 Department of Chemical Engineering, Faculty of Industrial Engineering, Universitas Pembangunan Nasional “Veteran” Yogyakarta, Yogyakarta 55283, Indonesia; 3 Department of Polymer Science and Engineering, Pusan National University, Busan 46241, Republic of Korea; 4 BB21+ Project Team, Pukyong National University, Busan 48547, Republic of Korea

**Keywords:** isosorbide-based polycarbonate (ISB-PC), cellulose nanocrystals (CNCs), nanocomposites, soil burial, mechanical property

## Abstract

Recently, the development of biomass-based polymers has attracted a great deal of attention as a sustainable alternative to petroleum-based plastics. However, the relatively limited mechanical properties of these bio-derived polymers often require reinforcement through the inclusion of nanofillers, while the environmental durability of such nanocomposites remains insufficiently understood. In this regard, we prepared nanocomposites composed of isosorbide-based polycarbonate (ISB-PC) and surface-modified cellulose nanocrystals (CNCs), and examined the effect of nanofillers on the mechanical properties and their retention under humid soil exposure. Interestingly, the initial reinforcement effect provided by CNCs rapidly decreased during the early stage of exposure, and the mechanical properties of the ISB-PC composites eventually converged toward those of pristine ISB-PC within a relatively short time. Based on the time-dependent changes in mechanical properties and mass loss, we propose that the mechanical deterioration was associated with physical changes in the polymer matrix, accompanied by the formation of macroscale cavities within the films. Our results suggest that initial reinforcement does not necessarily ensure sustained mechanical performance under humid soil exposure.

## 1. Introduction

Isosorbide-based polycarbonate (ISB-PC) has been investigated as a sustainable alternative to conventional petroleum-based polycarbonate, driven by the demand for high-performance engineering plastics derived from renewable resources [1,2,3,4,5,6,7,8,9,10,11]. Due to the rigid bicyclic isosorbide structure, ISB-PC exhibits high glass transition temperature, high stiffness, and optical transparency [1,2,3,4,5,6,7,8]. These characteristics make this polymer a promising candidate for various engineering applications [1,5,8,9]. However, ISB-PC generally suffers from inherent brittleness compared to conventional aromatic polycarbonates [3,4,6,10]. Furthermore, the susceptibility of ISB-PC to prolonged environmental exposure raises concerns about the retention of its mechanical performance [11]. These limitations indicate the need for further studies on the mechanical performance and environmental durability of ISB-PC.

To address the mechanical limitations of ISB-PC, cellulose nanocrystals (CNCs) have been investigated as sustainable reinforcing fillers owing to their high stiffness, low density, and renewable origin [8,9,10]. However, effective incorporation of CNC into hydrophobic polymer matrix such as ISB-PC still remains challenging because the intrinsic hydrophilicity of CNC often leads to poor dispersion and weak interfacial adhesion between the filler and the matrix [8,9,10,12,13,14,15,16,17,18,19]. To mitigate this incompatibility, various CNC surface modification strategies, including esterification, acetylation, polymer grafting, and silane-based treatments, have been explored [15,16,17,18,19,20,21,22,23,24]. Among these approaches, silane coupling agents are particularly attractive because they covalently modify the surface hydroxyl groups on CNC while introducing functional moieties compatible with polymer matrices [15,16,19,20,21,22,23]. In this context, 3-aminopropyltriethoxysilane (APTES) has been widely employed to introduce amine groups onto CNC surfaces, thereby improving dispersion and interfacial adhesion in polymer composites [15,16,19].

However, most previous studies on CNC-reinforced polymer nanocomposites, including ISB-PC-based systems, have focused primarily on initial mechanical reinforcement, while the retention of mechanical performance under environmental exposure remains largely unexplored. Given that a substantial fraction of plastic waste is disposed of in landfills or enters terrestrial ecosystems [25], understanding mechanical property retention under soil-contact conditions is necessary. In such environments, moisture, temperature changes, and microbial activity may promote physical disintegration, chemical degradation, interfacial weakening, and loss of matrix integrity [26]. Previous studies have reported microcavity formation and mechanical deterioration in polycarbonate-based materials exposed to water at elevated temperatures. For example, steam sterilization and hot-water exposure have been shown to induce microcavities associated with embrittlement in polycarbonate [27,28]. Similarly, voids and cracks have been observed in polycarbonate composites during hydrothermal aging, accompanied by changes in mechanical properties [29]. Such structural damage may be particularly relevant to solvent-cast films because microscopic inhomogeneities introduced during solvent casting can increase susceptibility to environmental exposure and weaken interfacial interactions [30,31]. The importance of the microstructure-property relationship has also been demonstrated by Wang et al., who reported that phase separation in solvent-cast PCL/PSBMA films significantly affected their tribological behavior [32]. In this regard, we investigate solvent-cast ISB-PC/CNC nanocomposite films containing pristine and APTES-modified CNCs to examine how initial reinforcement is retained or lost under soil exposure. These results could provide insight into the environmental durability of these composites.

## 2. Materials and Methods

### 2.1. Materials

Cellulose nanocrystals (CNCs) were purchased from CelluForce Inc. (Montreal, QC, Canada). Isosorbide-based polycarbonate (ISB-PC) was provided by Samyang Corp. (Seoul, Korea). 3-aminopropyltriethoxysilane (APTES) was obtained from Sigma-Aldrich (St. Louis, MO, USA). Chloroform (HPLC grade) and ethanol were purchased from Daejung Chemicals & Metals Co., Ltd. (Siheung, Korea) and Duksan Pure Chemicals Co., Ltd. (Ansan, Korea), respectively. Standard compost soil was purchased from Abnexo Co., Ltd. (Uiwang, Korea). All reagents were used as received.

### 2.2. Surface Modification of Cellulose Nanocrystals

The surface of CNCs was modified with APTES. For this purpose, 2.5 g of CNCs was dispersed in a mixture of ethanol (40 g) and deionized water (10 g) by bath sonication for 15 min, followed by stirring for 2 h. Separately, 5 g of APTES was added to a mixture of ethanol (8 g) and deionized water (2 g), and the pH was adjusted to ~4.0 using acetic acid (1 wt% in H_2_O). The two solutions were mixed, stirred for 2 h, and centrifuged at 2500 rpm for 20 min. The precipitate was washed with deionized water and re-dispersed in H_2_O. This centrifugation-based washing step was performed twice. The resulting dispersion was freeze-dried for 24 h and subsequently heated at 120 °C for 2 h under vacuum to obtain APTES-CNCs.

### 2.3. Preparation of ISB-PC Composite Films

Before preparing the ISB-PC/CNC composite films, chloroform was dried over activated 3 Å molecular sieves for 48 h. Then, CNCs were first dispersed in chloroform by bath sonication for 30 min to obtain a homogeneous dispersion. Subsequently, pre-determined amounts of ISB-PC and chloroform were added to the above CNC dispersion. In this step, the concentration of ISB-PC polymer was fixed at 10 wt% relative to the total mass of the solution, while the CNC content was adjusted to 1.0 wt% relative to the mass of ISB-PC. The resulting solutions were stirred for 6 h and poured into round Teflon molds (diameter = 10 cm). After solvent evaporation at 30 °C for 3 days, the cast films were further dried in a vacuum oven at 50 °C for 24 h. ISB-PC/APTES-CNC composite films were prepared using the same procedure, with APTES-CNCs replacing pristine CNCs. Finally, the films were cut into dumbbell-shaped specimens with an overall length of 60 mm, a gauge length of 15 mm, a gauge width of 4 mm, and a grip width of 15 mm for subsequent mechanical testing and soil burial experiments.

### 2.4. Soil Burial Test

Specimens of three compositions (ISB-PC, ISB-PC/CNC, and ISB-PC/APTES-CNC) were buried in compost-filled beakers at 58 °C to simulate thermophilic composting conditions. Dumbbell-shaped specimens were sandwiched between two 5 cm thick compost layers in glass beakers (diameter = 131 mm) containing approximately 700 g of compost. For each exposure period (7, 14, and 21 days), five specimens of each composition were distributed across two beakers. Each beaker contained two or three specimens per composition, and six beakers were used in total. The beakers were covered with perforated aluminum foil, and the pH of the compost was maintained at 7. Soil moisture was maintained by daily spraying of deionized water, with soil moisture meter (LY201) readings of 50–55%. Samples were retrieved after 7, 14, and 21 days of soil exposure, rinsed with deionized water, and dried at 40 °C for 24 h before further analysis. Mass loss (ML) was calculated using ML (%) = [(*w*_0_ − *w_t_*)/*w*_0_] × 100, where *w*_0_ and *w_t_* are the weight of specimens before and after soil burial.

### 2.5. Characterization

Fourier-transform infrared (FT-IR) spectra of the composites were obtained using a Fourier-transform infrared spectrometer Spectrum 3 (PerkinElmer Inc., Shelton, CT, USA). Dynamic Light Scattering (DLS) measurements were conducted with Zetasizer Nano ZS90 instrument (Malvern Panalytical Ltd., Malvern, UK). CNC and APTES-CNC were dispersed in deionized water or chloroform at a concentration of 0.01 wt% by bath sonication (30 min); each sample was measured three times and the measurements were averaged. The DLS results were fitted with a Gaussian function to obtain the mean hydrodynamic diameter and the standard deviation of the distribution. UV–vis spectrophotometer Cary 8454 (Agilent Technologies Inc., Santa Clara, CA, USA) was used to measure the optical transmittances of the prepared samples. Field-emission scanning electron microscopy (FE-SEM) images were acquired using SIGMA560 microscope (ZEISS, Oberkochen, Germany) with an acceleration voltage of 3 kV. For CNC and APTES-CNC, aqueous suspensions (0.1 wt%) were deposited onto silicon wafers and dried under ambient conditions. Cross sections of the composite films were prepared by immersing the films in liquid nitrogen for approximately 30 s, followed by immediate fracture. All samples were sputter-coated with Pt prior to observation. X-ray photoelectron spectroscopy (XPS) measurements were conducted using an AXIS SUPRA (KRATOS Analytical Ltd., Manchester, UK). Mechanical properties were measured using an Instron 34SC-1 (Instron, Norwood, MA, USA) with a 1 kN load cell at a crosshead speed of 1 mm min^−1^.

## 3. Results and Discussion

### 3.1. Surface Modification and Characterization of Cellulose Nanocrystals

**Figure 1:**
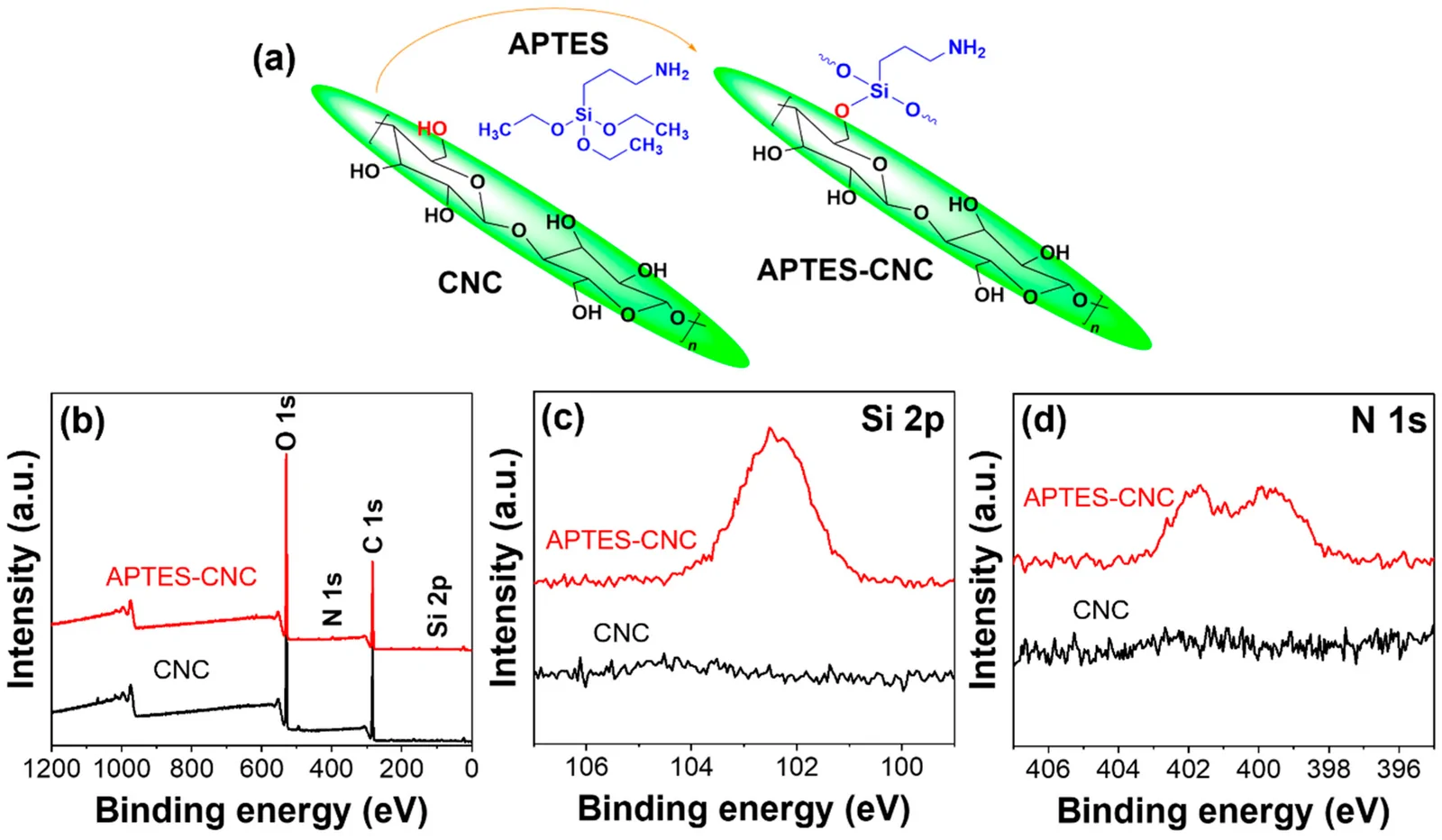
(**a**) Schematic illustration of CNC surface modification with APTES; (**b**–**d**) XPS spectra of unmodified CNC and APTES-CNC: (**b**) survey scan; (**c**) Si 2p region; and (**d**) N 1s region.

The incorporation of APTES-modified CNCs (APTES-CNCs) into ISB-PC was investigated as a strategy to enhance the mechanical performance of ISB-PC-based nanocomposites. Figure 1a depicts the proposed molecular structure of APTES-CNC, in which APTES reacts with surface hydroxyls of CNC to form Si-O-C covalent bonds through silane condensation [15,16,22,23]. The introduced aminopropyl moieties can reduce the surface polarity of CNCs and improve their dispersion within the ISB-PC matrix [15,16,19]. The successful surface modification of CNCs was confirmed by X-ray photoelectron spectroscopy (XPS) analysis (Figure 1b–d). Both unmodified CNC and APTES-CNC exhibit strong C 1s and O 1s peaks at 286.4 and 532.8 eV, respectively, which are consistent with the cellulose structure [21,23,33]. Compared with unmodified CNC, APTES-CNC showed additional Si 2p and N 1s peaks, indicating the successful introduction of APTES onto the CNC surface. In the high-resolution Si 2p spectrum (Figure 1c), the peak at 102.3 eV could be attributed to Si–O–C bonds formed through silanization of the CNC surface with APTES and/or Si–O–Si bonds formed through self-condensation of APTES, as their binding energies overlap in this region [34]. The N 1s spectrum (Figure 1d) shows two components at 399.6 eV and 401.7 eV, assigned to –NH_2_ and –NH_3_^+^, respectively [35,36].

### 3.2. Morphology and Dispersion of Cellulose Nanocrystals

**Figure 2:**
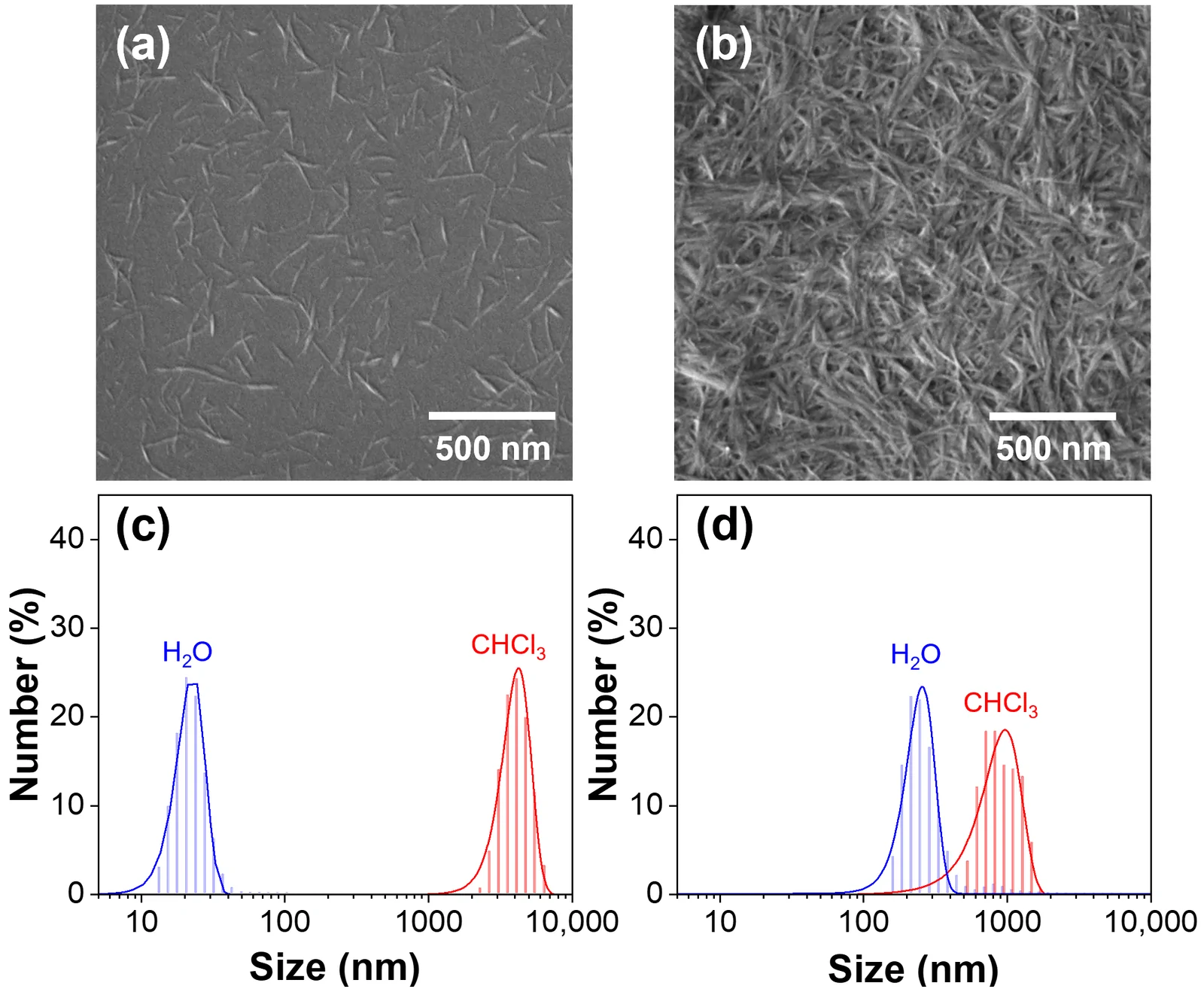
(**a**,**b**) FE-SEM images of (**a**) pristine CNC and (**b**) APTES-CNC. (**c**,**d**) DLS results of (**c**) pristine CNC and (**d**) APTES-CNC dispersed in H_2_O (blue) and chloroform (red).

Following surface chemical characterization, morphological differences between unmodified CNC and APTES-CNC were examined using field-emission scanning electron microscopy (FE-SEM). As shown in Figure 2a, the SEM image of unmodified CNC exhibits well-defined rod-like nanocrystals with minimal agglomeration, reflecting good dispersion in aqueous solution. This behavior can be attributed to the abundant surface hydroxyl groups and the residual sulfate ester groups resulting from the sulfuric acid hydrolysis of cellulose [37]. These functional groups promote favorable interactions with water molecules and provide sufficient electrostatic repulsion to maintain a stable, non-aggregated state in an aqueous environment. In contrast, APTES-CNC exhibited a more densely packed morphology (Figure 2b), which would be ascribed to reduced surface hydrophilicity after APTES modification.

Dynamic light scattering (DLS) analysis further reveals the effect of surface modification on the dispersion behavior of CNC (Figure 2c) and APTES-CNC (Figure 2d). It is worth noting that the particle sizes obtained from DLS correspond to hydrodynamic diameters rather than the actual physical dimensions of rod-like CNCs or their aggregates. Therefore, these values are used here as comparative indicators of aggregation behavior. In water, both CNCs and APTES–CNCs remain well dispersed, with average particle sizes of approximately 23 ± 5 nm and 260 ± 57 nm, respectively. The larger hydrodynamic size of APTES-CNC can be attributed to reduced surface hydrophilicity after surface modification, suggesting the formation of small clusters while maintaining a stable suspension. When the solvent was changed to chloroform, which was used as the processing solvent for ISB-PC, unmodified CNC exhibited severe macroscale aggregation with a hydrodynamic size of approximately 4230 ± 950 nm. In contrast, APTES modification significantly mitigated this aggregation, reducing the hydrodynamic size to approximately 970 ± 295 nm.

### 3.3. Chemical and Optical Characterization of ISB-PC Composite Films

**Figure 3:**
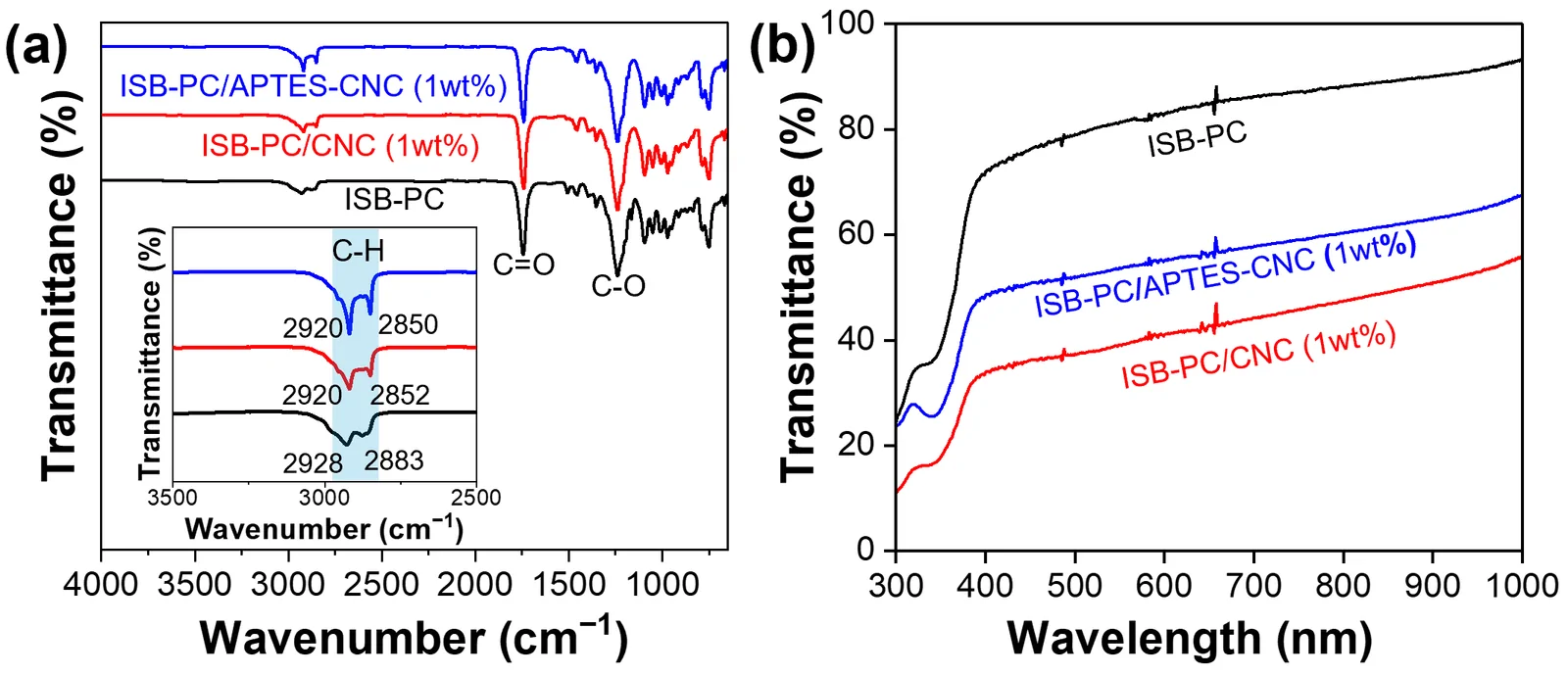
(**a**) FT-IR spectra and (**b**) UV–Vis transmittance spectra of neat ISB-PC (black), ISB-PC/CNC (red), and ISB-PC/APTES-CNC (blue) composite films.

After verifying the dispersibility of CNC and APTES-CNC in chloroform, each filler was dispersed with ISB-PC in chloroform and then composite films were prepared by solvent casting. In the composites, the filler content was fixed at 1.0 wt% relative to the mass of ISB-PC. The incorporation of CNC and APTES-CNC was analyzed by FT-IR to examine changes in the local chemical environment of the ISB-PC matrix (Figure 3a). While the characteristic carbonyl (C=O) stretching band of ISB-PC at ~1740 cm^−1^ remained largely unaffected across all samples, certain changes were observed in the C–H stretching region. Neat ISB-PC exhibited aliphatic C–H stretching bands at 2928 cm^−1^ and 2883 cm^−1^ [3,6,7]. Upon filler incorporation, these bands shifted toward lower wavenumbers (~2920 cm^−1^ and 2850 cm^−1^) and became sharper. Although aliphatic C–H vibrations from the APTES moiety may overlap with this region, their direct contribution to the overall FT-IR intensity is expected to be limited because the APTES-CNC content was only 1 wt% relative to the mass of polymer matrix. Therefore, the observed shift and sharpening are more likely associated with changes in the local chain environment of ISB-PC induced by the incorporated fillers. Notably, these changes were more pronounced for ISB-PC/APTES-CNC than for ISB-PC/CNC, possibly associated with improved compatibility between APTES-modified CNC and the ISB-PC matrix.

Next, the optical transmittance of the composite films was evaluated using UV–Vis spectroscopy (Figure 3b). The neat ISB-PC film exhibited the highest transmittance across the visible region (black curve), reaching approximately 81% at 550 nm, a representative wavelength for visible-light transmittance [38]. In contrast, the incorporation of unmodified CNC resulted in a pronounced decrease in optical transmittance (red curve). Specifically, the ISB-PC composite containing 1.0 wt% CNC showed a sharp reduction to 39% at 550 nm, which can be attributed to strong light scattering caused by CNC aggregation within the polymer matrix. This observation is consistent with the aggregation tendency revealed by the DLS analysis. By comparison, the ISB-PC composite containing 1.0 wt% APTES-CNC exhibited a higher transmittance of 54% at 550 nm (blue curve), indicating that APTES surface modification suppressed CNC aggregation and thereby reduced scattering losses. Nevertheless, the transmittance of ISB-PC/APTES-CNC remains lower than that of neat ISB-PC, suggesting the presence of partially aggregated fillers.

### 3.4. Mechanical Properties Under Humid Soil Exposure

**Figure 4:**
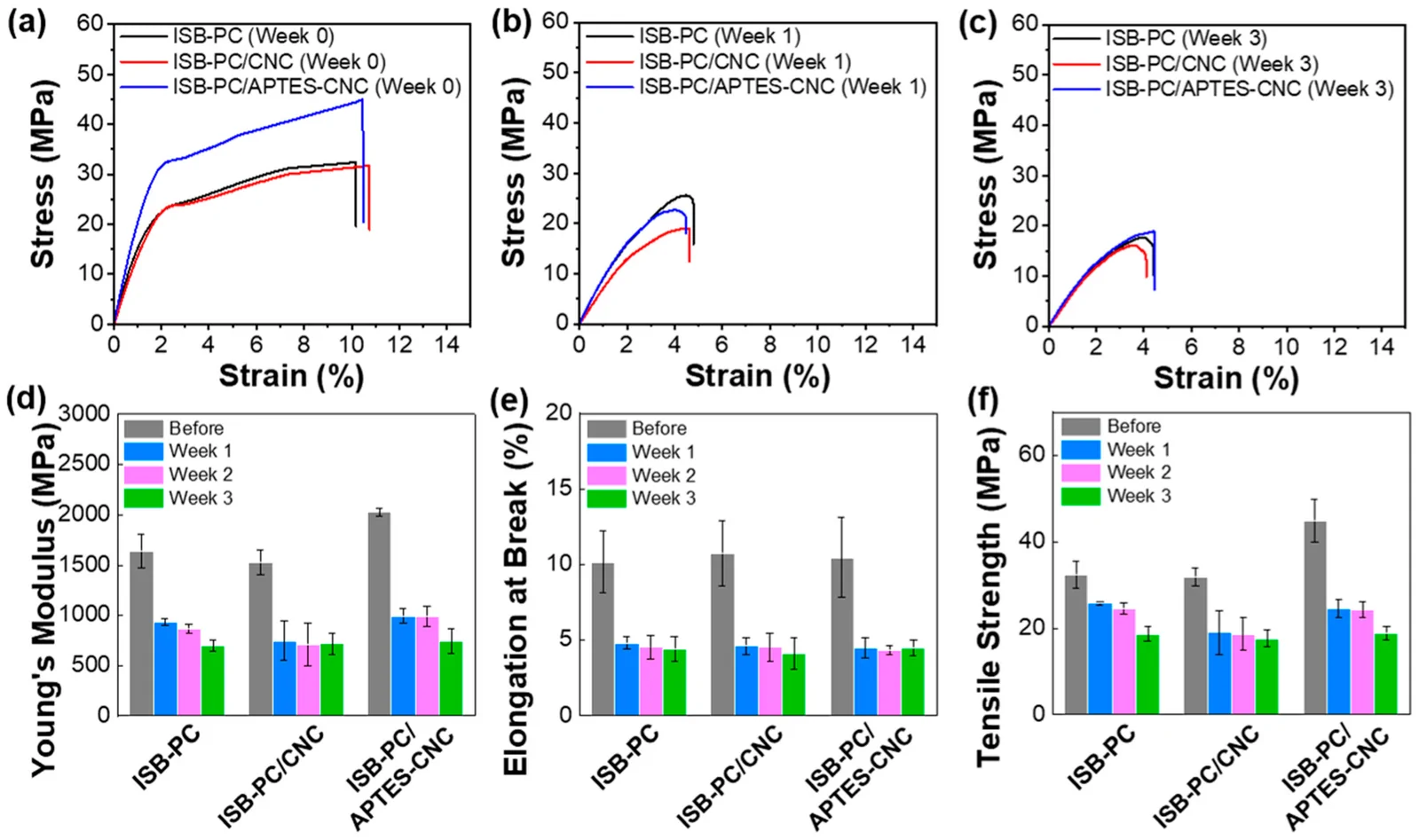
Mechanical properties of neat ISB-PC, ISB-PC/CNC (1.0 wt%) and ISB-PC/APTES-CNC (1.0 wt%) composite films before and after soil burial. (**a**–**c**) Stress–strain curves: (**a**) as-prepared, (**b**) after 1 week, and (**c**) after 3 weeks of soil burial. (**d**–**f**) Changes in (**d**) Young’s modulus, (**e**) elongation at break, and (**f**) tensile strength of the films during soil burial. Error bars represent standard deviation of measurements from five specimens tested under each condition.

Based on the dispersion characteristics discussed above, the mechanical properties of ISB-PC composites containing CNC or APTES-CNC were evaluated by tensile testing. As shown in Figure 4a, neat ISB-PC exhibited a Young’s modulus of 1640 MPa, a tensile strength of 32.4 MPa, and an elongation at break of 10.2%. The incorporation of unmodified CNCs led to a slight reduction in tensile modulus and tensile strength to 1524 MPa and 31.8 MPa, respectively. In contrast, the ISB-PC composites containing APTES-CNC showed improved mechanical performance, with a Young’s modulus of 2027 MPa and a tensile strength of 44.9 MPa. This result suggests that APTES modification enhanced filler–matrix compatibility, enabling more effective stress transfer across the interface. Subsequently, the specimens were buried under humid soil conditions, and their mechanical property retention was evaluated over three weeks of soil exposure. Figure 4b,c show representative tensile stress–strain curves of the composites after 1 and 3 weeks of exposure, respectively. For a clearer comparison, the changes in Young’s modulus, tensile strength, and elongation at break during soil exposure are summarized in Figure 4d–f. As confirmed from Figure 4, all samples exhibited a pronounced decrease in mechanical performance during the first week of exposure, followed by a slower decline over the subsequent weeks. Although nanocellulose reinforcement improved the initial mechanical properties, the differences between reinforced and unreinforced samples gradually diminished during soil exposure. After three weeks, all samples exhibited comparable mechanical properties, indicating that the initial reinforcement effect was largely lost under humid soil exposure. Specifically, neat ISB-PC exhibited a Young’s modulus of 695 MPa, a tensile strength of 18.7 MPa, and an elongation at break of 4.4%. To examine whether the differences in mechanical properties among the three materials were statistically significant, a one-way analysis of variance (ANOVA) was performed. The results showed significant differences in Young’s modulus and tensile strength before soil burial (*p* < 0.05), but these differences were no longer statistically significant after three weeks of exposure (*p* > 0.05) (Table 1). It is worth noting that similar trends were observed across three independent experimental runs, despite minor variations in the measured mechanical properties.

### 3.5. Mass-Loss Behavior

The mass of the ISB-PC-based films was also measured at weekly intervals during soil exposure. As shown in Table 2, all samples exhibited rapid mass loss during the first week, with values of 7.6% for pristine ISB-PC, 6.9% for ISB-PC/CNC, and 7.2% for ISB-PC/APTES-CNC. Thereafter, only minor additional mass loss was observed, reaching approximately 8.2–8.4% after three weeks. This plateau-like behavior suggests that the mass loss was not primarily associated with continuous chemical degradation of the polymer backbone. This mass-loss behavior paralleled the trends in mechanical properties shown in Figure 4. In separate control experiments, the specimens were either washed using the same procedure without soil burial or immersed in water at room temperature for 1 day. However, no measurable weight change was observed. These control results support that the observed mass loss was associated with the soil-exposure condition rather than sample handling, washing, or short-term water exposure. Nevertheless, it needs to be noted that the mass-loss behavior alone cannot exclude possible contributions from chemical degradation. In this sense, additional characterization would be necessary to further clarify the relative contributions of physical and chemical processes to the observed mass loss and deterioration of mechanical properties.

### 3.6. Morphological Changes Under Humid Soil Exposure

**Figure 5:**
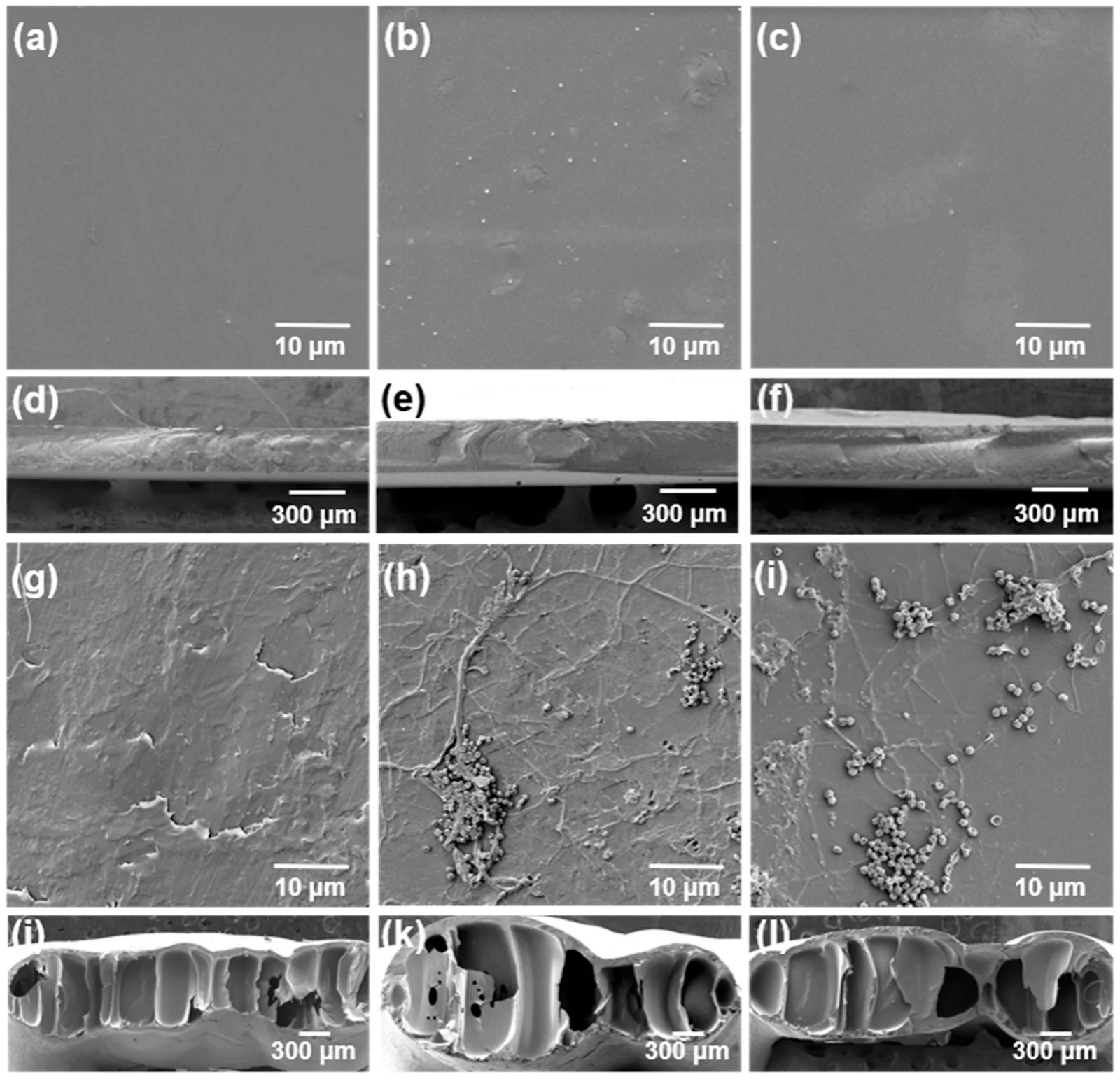
FE-SEM images of ISB-PC and ISB-PC composite films before and after soil burial: (**a**–**c**) surface images and (**d**–**f**) cross-sectional images before soil burial; (**g**–**i**) surface images and (**j**–**l**) cross-sectional images after 3 weeks of soil burial. (**a**,**d**,**g**,**j**) Neat ISB-PC, (**b**,**e**,**h**,**k**) ISB-PC/CNC (1 wt%), and (**c**,**f**,**i**,**l**) ISB-PC/APTES-CNC (1 wt%).

To further examine the morphological changes associated with mechanical deterioration and mass loss during soil exposure, FE-SEM analysis was performed on the surfaces and cross sections of the ISB-PC-based films before and after burial (Figure 5). Before soil burial, the surfaces of all films appeared generally homogeneous (Figure 5a−c). For the ISB-PC/CNC film (Figure 5b), small white dot-like features were observed, likely attributable to localized CNC aggregates. These features were less pronounced in the ISB-PC/APTES-CNC film (Figure 5c), suggesting that APTES modification improved the dispersion state of CNCs within the ISB-PC matrix. Cross-sectional FE-SEM images also showed relatively uniform morphologies without obvious cavities or macroscopic defects (Figure 5d–f). After three weeks of soil exposure, the film surfaces became noticeably rougher and more heterogeneous (Figure 5g–i). In particular, both ISB-PC/CNC and ISB-PC/APTES-CNC films exhibited localized spherical or colony-like features, which may be associated with microbial attachment [39,40,41]. However, their identity and contribution to the observed mass loss and deterioration of mechanical properties remain unresolved at this stage. More pronounced morphological transformations were observed in the cross-sectional FE-SEM images. As shown in Figure 5j–l, macrovoid-like cavities were formed within all samples, in clear contrast to the relatively uniform cross sections before soil burial. Given that the films were prepared by solvent casting, microscopic heterogeneities such as microvoids and/or residual stresses may have existed within the as-prepared films [30]. We therefore propose that, during exposure to the humid soil environment at 58 °C, absorbed water could increase local chain mobility and induce swelling of the ISB-PC matrix [11]. Such moisture-induced changes may have promoted the growth and coalescence of pre-existing microscopic heterogeneities, eventually leading to the formation of internal void structures. It is worth noting that cavity formation has previously been reported in polycarbonate-based materials under hydrothermal conditions, including steam sterilization and hot-water immersion [27,28]. Although the exposure conditions and cavity formation mechanisms may differ, the present observations suggest that internal cavities can also develop under humid soil exposure.

In addition, the films became visibly whitened after one week of soil burial, and small void-like regions appeared beneath the film surface and gradually enlarged with increasing exposure time. These visual changes are consistent with the macrovoid-like cavities observed in the cross-sectional SEM images. Such internal defects would be associated with the rapid mass loss observed during the early stage of soil exposure, which may involve the extraction of low-molecular-weight components, local fragmentation of the films, or other processes. Furthermore, the formation of macrovoid-like cavities would reduce the structural integrity of the ISB-PC matrix and weaken the effective load-bearing contribution of the nanocellulose fillers. As matrix-level defects developed, the initial reinforcing effect of CNC and APTES-CNC would be progressively lost, and the mechanical properties become primarily governed by the ISB-PC matrix.

## 4. Conclusions

This study investigated the mechanical reinforcement and early-stage durability of ISB-PC nanocomposites reinforced with cellulose nanocrystals under humid soil exposure. Compared with unmodified CNC, APTES-CNC exhibited improved dispersion in the ISB-PC matrix, resulting in enhanced initial mechanical properties. However, under humid soil exposure, the mechanical properties of all samples decreased markedly during the first week and declined more gradually over the remaining exposure period. Eventually, the mechanical properties of the ISB-PC nanocomposites approached those of pristine ISB-PC. Similarly, most of the mass loss occurred during the first week, with only minor additional loss during continued exposure. These parallel trends, together with the macroscale cavities observed in all films after soil exposure, suggest that the early-stage deterioration is associated with moisture-induced physical changes in the solvent-cast films, although contributions from chemical changes, such as hydrolysis or chain scission, cannot be excluded. These results demonstrate that initial mechanical reinforcement does not necessarily translate into sustained mechanical performance under humid soil exposure.

## Figures and Tables

**Table 1:** Statistical analysis of mechanical properties of ISB-PC and its composites.

Mechanical Property	Before Soil Burial (*p*-Value)	After 3 Weeks (*p*-Value)
Young’s Modulus	0.005	0.679
Tensile Strength	<0.001	0.298

*p*-values were obtained from one-way ANOVA (*n* = 5 per group; significance level = 0.05).

**Table 2:** Mass loss of ISB-PC and nanocomposites.

Sample	Week 1 [%]	Week 2 [%]	Week 3 [%]
ISB-PC	7.6 ± 0.7	8.0 ± 0.6	8.2 ± 0.4
ISB-PC/CNC (1.0 wt%)	6.9 ± 0.3	8.2 ± 0.4	8.3 ± 0.7
ISB-PC/APTES-CNC (1.0 wt%)	7.2 ± 0.4	7.9 ± 0.6	8.4 ± 0.6

Values are presented as mean ± standard deviation from five specimens tested under each condition.

## Data Availability

The data that support the findings of this study are available from the corresponding author upon reasonable request.

## Abbreviations

The following abbreviations are used in this manuscript:

APTES	3-aminopropyltriethoxysilane
CNC	Cellulose nanocrystal
DLS	Dynamic light scattering
FE-SEM	Field-emission scanning electron microscopy
FT-IR	Fourier-transform infrared
ISB-PC	Isosorbide-based polycarbonate
XPS	X-ray photoelectron spectroscopy
